# Prevalence of Nonalcoholic Fatty Liver Disease at a Tertiary Care Center in Saudi Arabia

**DOI:** 10.7759/cureus.47896

**Published:** 2023-10-29

**Authors:** Ghada Hussein, Aljoharah A Al Saud, Abdulelah A Bashandi, Mohammed M Almousallam, Reem M AlShihri, Osama M Almousallam, Ibrahim M Binsalamah, Yaser Alendijani

**Affiliations:** 1 Family Medicine, King Faisal Specialist Hospital and Research Centre, Riyadh, SAU; 2 Family Medicine and Polyclinics, Alfaisal University College of Medicine, Riyadh, SAU; 3 Family Medicine and Polyclinics, King Faisal Specialist Hospital and Research Centre, Riyadh, SAU

**Keywords:** nonalcoholic fatty liver disease (nafld), obesity, family medicine, fatty liver, fib-4, nafld

## Abstract

Objectives: To determine the prevalence of nonalcoholic fatty liver disease (NAFLD) in patients who received abdominal imaging and to assess the clinical and metabolic characteristics of NAFLD.

Methods: This is a retrospective study of 500 family medicine patients (aged 18 years and older) who completed abdominal imaging at King Faisal Specialist Hospital & Research Centre, Riyadh, Saudi Arabia, from January 2016 through June 2020.

Results: The patients enrolled had a mean age of 49.41 ± 14.80 years, with 300 females and 349 of Saudi nationality. The mean body mass index (BMI) was 29.43 ± 6.61 kg/m^2^, while 373 of the enrolled subjects were either overweight or obese. Half of our patients had some form of fatty liver in the imaging results. Regarding chronic medical conditions, 33.4%, 31.4%, and 29.4% had a history of hypertension, type 2 diabetes mellitus (DM2), and dyslipidemia, respectively. The mean Fibrosis-4 (FIB-4) index was 0.94 ± 0.72. Body mass index was higher among fatty liver patients (p = 0.001). Hypertension, coronary artery disease, dyslipidemia, and DM2 were more common in the fatty liver group.

Conclusion: Our findings reiterate the significance of obesity and the coexistence of cardiovascular risk factors in NAFLD. Further studies are needed to corroborate and expand our findings, enabling more refined strategies for the prevention, risk prediction, early detection, and management of NAFLD.

## Introduction

Nonalcoholic fatty liver disease (NAFLD) is defined as the accumulation of excess fat in the liver in the absence of other causes [1]. The spectrum of NAFLD encompasses fatty liver changes on abdominal imaging, some normal liver function tests, and elevated liver function tests, which are associated with advanced liver cirrhosis [2].

The worldwide prevalence of NAFLD is 25% [1], with the highest prevalence being in the Middle East at 32% [3]. The most important risk factors for NAFLD are being overweight, obesity, and type 2 diabetes (DM2) [4]. One-third of the Middle East is obese, and the prevalence of DM2 is increasing rapidly in Saudi Arabia [2]. Therefore, diagnosing NAFLD early and initiating prompt treatment is critical for decreasing complications and mortality [5].

Nonalcoholic fatty liver disease is associated with morbidity and mortality. Death due to cardiovascular disease is attributed to NAFLD [5]. Nonalcoholic fatty liver disease can lead to liver-related mortality and morbidity due to hepatocellular carcinoma with or without cirrhosis [3].

A nonalcoholic fatty liver disease diagnosis can be confirmed through invasive or noninvasive testing. Liver biopsies are the gold standard for assessing liver fibrosis; however, they are not recommended for all patients with NAFLD due to the risk of complications. Indeed, many patients are unwilling to undergo these procedures [3]. Increased liver fat is usually seen on an abdominal ultrasound (US) when more than 20% of the hepatocytes are affected, while non-contrast computerized tomography (CT) is more specific than the US for NAFLD [6]. Magnetic resonance imaging (MRI) is the most sensitive modality for evaluating hepatic steatosis. As little as 5% steatosis can be detected [6]. Furthermore, biochemical markers-aminotransferases, bilirubin, and ferritin-and metabolic markers-glycated hemoglobin (HbA1C) and lipids [3]-are used as predictors of NAFLD diagnoses [5].

The Fibrosis-4 (FIB-4) index is a validated score that estimates liver fibrosis without a biopsy, calculated from age, serum alanine aminotransferase (ALT), aspartate aminotransferase (AST), and platelet count. The FIB-4 index should be used as a screening tool for NAFLD in high-risk groups. A person with a FIB-4 index <1.30 is considered at low risk for developing liver fibrosis [7]. A person is at high risk with a FIB-4 index >2.67, as per the fibrosis risk stratification, and, thus, should be referred to a liver specialist and recommended a liver biopsy [7]. Persons of indeterminate risk with a FIB-4 index of 1.3-2.67 need further assessment of liver stiffness and referral to a liver specialist [7].

According to the American College of Gastroenterology, normal ALT levels should be 29 to 33 U/L for men and 19 to 25 U/L for women, while elevated levels need further assessment [8]. Hepatocellular injury may be suggested by elevated AST and/or ALT, alkaline phosphatase, and bilirubin [8]. Alanine aminotransferase is a more specific marker for hepatocellular cell injury, as it is mainly present in the liver [8].

There is an urgent need for further evaluation of NAFLD since most patients are asymptomatic, and early detection is warranted for this highly prevalent disease to improve health outcomes. The primary objective was to assess the prevalence of NAFLD in family medicine patients who received abdominal imaging. The secondary objective is to evaluate the clinical and metabolic characteristics of NAFLD.

## Materials and methods

The retrospective review included the Integrated Clinical Information System (ICIS) database of the electronic health records of patients aged 18 or older seen at King Faisal Specialist Hospital and Research Centre Family Medicine clinics from January 1, 2016-June 30, 2020, who had abdominal imaging (i.e., abdominal ultrasound, CT scan of the abdomen, or MRI of the abdomen). The data collected included demographics, medical history, BMI, tobacco use, blood pressure, and lab results within three months of imaging, including fasting glucose, HbA1C, AST, ALT, platelet, ALP (alkaline phosphatase), bilirubin, ferritin, lipid profile, creatinine, GFR (glomerular filtration rate), TSH (thyroid stimulating hormone), uric acid, PT (prothrombin time), INR (international normalized ratio), and liver imaging findings. The FIB-4 index was calculated using the formula with lab values and age from the ICIS system.

Patients were excluded if they have coexisting liver disease, hepatitis B or C, alcohol use or history, autoimmune liver disease, viral hepatitis, pregnancy, hepatocellular carcinoma, secondary causes, or a history of bariatric surgery. Patients were also excluded if they were taking methotrexate, valproic acid, amiodarone, and corticosteroids.

All data were analyzed using the IBM SPSS statistical software package, version 20.0 (IBM Corp., Armonk, NY). Descriptive statistics for the categorical variables were presented as frequencies and percentages. The continuous variables were summarized as the mean ± standard deviation (SD). Inferential statistics was performed on continuous variables using the independent t-test to measure the statistical differences between patients diagnosed with NAFLD and those who were not. Conversely, categorical variables were compared using the chi-square test and Fisher’s exact test. Univariate logistic regression was performed to examine the associations between NAFLD and the other factors. The significance level was set at 0.05 with a 95% confidence interval (CI). Ethical approval was obtained from the Research Ethics Committee at King Faisal Specialist Hospital and Research Centre, Riyadh, Saudi Arabia, on January 8, 2023 (approval number: RAC 2221264), and a waiver of signed informed consent was granted.

## Results

Five hundred patients were included in the analysis (Table 1).

**Table 1:** Descriptive information of the study population

Variables	n (%)
Age, in years (Mean ± SD)	49.41 ± 14.80
< 21	7 (1.4)
21–30	50 (10)
31–40	87 (17.4)
41–50	114 (22.8)
51–60	140 (28)
61–70	61 (12.2)
> 70	41 (8.2)
Gender	
Female	300 (60)
Male	200 (40)
Nationality	
Saudi	349 (69.9)
Non-Saudi	150 (30.1)
Marital status	
Single	125 (25.2)
Married	345 (69.4)
Divorced	9 (1.8)
Widow/widower	18 (3.6)
BMI (Mean ± SD)	29.43 ± 6.61
Underweight	12 (2.4)
Normal weight	109 (22.1)
Overweight	176 (35.6)
Obese I	114 (23.1)
Obese II	48 (9.7)
Obese III	35 (7.1)
Smoking status	
No	445 (90.4)
Yes	47 (9.6)
Fatty liver based on physician diagnosis	
No	467 (93.4)
Yes	33 (6.6)
Fatty liver based on imaging results	
Normal	248 (49.6)
Fatty liver	67 (13.4)
Mild fatty liver	82 (16.4)
Mild to moderate fatty liver	9 (1.8)
Moderate fatty liver	52 (10.4)
Moderate to severe fatty liver	19 (3.8)
Severe fatty liver	21 (4.2)
Unclear classification	2 (0.4)
Medical history	
Hypertension	167 (33.4)
Coronary artery disease or myocardial infarction	20 (4)
Dyslipidemia	147 (29.4)
Type 2 diabetes mellitus	157 (31.4)
Hypothyroidism	67 (13.4)
FIB-4 index (Mean ± SD)	0.94 ± 0.72
Low	409 (81.8)
Indeterminate	78 (15.6)
High	13 (2.6)

The mean age of the patient population was 49.41 ± 14.80 years, with 300 (60%) females and 345 (69.4%) married. The mean body mass index was 29.43 ± 6.61 kg/m2, with the greatest number of patients being either normal weight or overweight. The majority of patients were non-smokers. Thirty-three (6.6%) patients had a fatty liver diagnosis that a physician documented in their medical chart under their past medical history. According to the imaging results, half of our patients had some form of fatty liver. The prevalence of fatty liver in the studied sample who completed imaging was 50.4% (252). The most common chronic medical conditions were hypertension, type 2 diabetes mellitus, and dyslipidemia. Most patients had a low FIB-4 index, with a mean FIB-4 index of 0.94 ± 0.72.

Patients with a fatty liver upon imaging had a statistically significant higher mean age than non-fatty liver patients. Gender, nationality, and marital status did not differ between the two groups (Table 2).

**Table 2:** Prevalence of sociodemographic factors and medical history among patients with NAFLD NAFLD: nonalcoholic fatty liver disease

Variables	Fatty liver based on imaging results		p-value
	No	Yes	
Age, in years	47.54 ± 16.17	51.25 ± 13.08	0.005
< 21	5 (2)	2 (0.8)	0.004
21–30	36 (14.5)	14 (5.6)	
31–40	52 (21)	35 (13.9)	
41–50	46 (18.5)	68 (27)	
51–60	61 (24.6)	79 (31.3)	
61–70	25 (10.1)	36 (14.3)	
> 70	23 (9.3)	18 (7.1)	
Gender			0.971
Female	149 (60.1)	151 (59.9)	
Male	99 (39.9)	101 (40.1)	
Nationality			0.261
Saudi	167 (67.6)	182 (72.2)	
Non-Saudi	80 (32.4)	70 (27.8)	
Marital status			0.368
Single	70 (28.5)	55 (21.9)	
Married	163 (66.3)	182 (72.5)	
Divorced	5 (2)	4 (1.6)	
Widow/widower	8 (3.3)	10 (4)	
BMI	27.28 ± 5.84	31.54 ± 6.65	0.001
Underweight	10 (4.1)	2 (0.8)	0.001
Normal weight	80 (32.7)	29 (11.6)	
Overweight	97 (39.6)	79 (31.7)	
Obese I	38 (15.5)	76 (30.5)	
Obese II	12 (4.9)	36 (14.5)	
Obese III	8 (3.3)	27 (10.8)	
Smoking status			0.137
No	216 (87.8)	229 (93.1)	
Yes	30 (12.2)	17 (6.9)	
Fatty liver diagnosis placed by physician in medical history			0.001
No	246 (99.2)	221 (87.7)	
Yes	2 (0.8)	31 (12.3)	
Medical history			
Hypertension	59 (23.8)	108 (42.9)	0.001
Coronary artery disease or myocardial infarction	4 (1.6)	16 (6.3)	0.010
Dyslipidemia	40 (16.1)	107 (42.5)	0.001
Type 2 diabetes mellitus	50 (20.2)	107 (42.5)	0.001
Hypothyroidism	32 (12.9)	35 (13.9)	0.746
FIB-4 index	0.94 ± 0.84	0.95 ± 0.58	0.824
Low	206 (83.1)	203 (80.6)	0.642
Indeterminate	37 (14.9)	41 (16.3)	
High	5 (2)	8 (3.2)	

Body mass index was greater among fatty liver patients (p=0.001). In terms of cardiovascular risk factors, hypertension, coronary artery disease, dyslipidemia, and type 2 diabetes mellitus were more common in the fatty liver group. Interestingly, the mean FIB-4 index and FIB-4 risk category were not significantly different between the two groups.

We also compared various laboratory parameters between fatty liver and non-fatty liver patients (Table 3).

**Table 3:** The mean difference in laboratory parameters among patients with NAFLD ALT: alanine aminotransferase; AST: aspartate aminotransferase; ALP: alkaline phosphatase; HDL-C: high-density lipoprotein cholesterol; LDL-C: low-density lipoprotein cholesterol; TSH: thyroid stimulating hormone; PT: prothrombin time; INR: international normalized ratio; NAFLD: nonalcoholic fatty liver disease

Parameters	Fatty liver based on imaging results		p-value
	No	Yes	
ALT	21.77 ± 20.41	33.32 ± 28.52	0.001
AST	20.99 ± 11.33	26.7 ± 20.06	0.001
Ferritin	98.69 ± 114.3	136.7 ± 173.34	0.081
ALP	75.15 ± 30.29	84.15 ± 45.4	0.010
Triglycerides	1.37 ± 0.8	1.64 ± 0.83	0.003
Cholesterol	4.53 ± 1.04	4.59 ± 0.93	0.588
HDL-C	1.33 ± 0.4	1.27 ± 0.43	0.166
LDL-C	2.97 ± 0.96	3.04 ± 0.86	0.508
Bilirubin	8.68 ± 7.74	8.62 ± 6.82	0.929
Creatinine	70.85 ± 24.86	71.94 ± 26.45	0.642
Uric acid	300.6 ± 96.47	322.16 ± 80.5	0.408
TSH	2.29 ± 2.15	2.47 ± 1.73	0.414
PT	14.25 ± 2.52	14.16 ± 2.48	0.820
INR	1.08 ± 0.21	1.05 ± 0.29	0.460

The ALT, AST, and ALP were statistically higher among fatty liver patients. Lipid profiles did not vary between fatty liver and non-fatty liver patients, except for triglycerides, which were statistically elevated in NAFLD patients (p = 0.003). Bilirubin, ferritin, creatinine, uric acid, TSH, PT, and INR levels did not diverge among the patients.

Stage III obesity was the most predictive factor for NAFLD in our patient population, followed by stage II obesity and stage I obesity, as shown in Table 4.

**Table 4:** Predictor’s factors of NAFLD ALT: alanine aminotransferase; AST: aspartate aminotransferase; ALP: alkaline phosphatase; NAFLD: nonalcoholic fatty liver disease

Variable	OR	95% CI	p-value
Age	1.017	(1.005–1.03)	0.005
BMI			
Underweight	0.552	(0.114–2.669)	0.460
Normal weight	Reference		
Overweight	2.247	(1.338–3.773)	0.001
Obese I	5.517	(3.101–9.818)	0.001
Obese II	8.276	(3.796–18.041)	0.001
Obese III	9.310	(3.8–22.81)	0.001
Hypertension (Yes/No)	2.403	(1.636–3.528)	0.001
Coronary artery disease or myocardial infarction (Yes/No)	4.136	(1.363–12.551)	0.012
Dyslipidemia (Yes/No)	3.837	(2.52–5.844)	0.001
Type 2 diabetes mellitus (Yes/No)	2.922	(1.962–4.352)	0.001
ALT	1.024	(1.014–1.034)	0.001
AST	1.030	(1.014–1.046)	0.001
ALP	1.007	(1.001–1.013)	0.014
Triglycerides	1.609	(1.159–2.233)	0.004

The odds ratio of fatty liver disease was 9.3 times higher among stage III obese patients compared to normal-weight patients. Among cardiovascular risk factors, coronary artery disease, dyslipidemia, type 2 diabetes mellitus, and hypertension were all predictive of NAFLD. Additionally, increased ALT, AST, ALP, and triglycerides were notable predictors of NAFLD.

## Discussion

This study investigated the demographic, clinical, and laboratory characteristics of a patient population to determine the potential predictors of NAFLD at a Saudi tertiary center. Studies regarding NAFLD in Saudi Arabia are limited. A recent meta-analysis by Alenezi et al. included eight studies and found a pooled prevalence rate of NAFLD of 16.8% in the Kingdom [9]. Additionally, the study reported a prevalence rate of 58% among patients with type 2 diabetes mellitus (9). Hence, it is crucial to identify the predictors of NAFLD among the Saudi population to allow early prevention and treatment.

In addition, obesity emerged as a highly significant predictor of NAFLD, with higher levels of obesity associated with a higher risk of the disease. This corroborates the established literature indicating that obesity is a prominent risk factor for NAFLD, demonstrating the importance of screening for NAFLD in high-risk populations and of weight management strategies in the prevention and management of the disease. Weight loss can improve glycemic control, reduce steatosis, inflammation, and hepatic injury, and enhance the quality of life in NAFLD patients [10]. Interestingly, a sizable portion of our patient population had a normal BMI, suggesting that, while obesity is a significant risk factor, NAFLD can occur irrespective of body weight, underscoring the complexity of the disease’s etiology.

Cardiovascular risk factors, such as hypertension, coronary artery disease, dyslipidemia, and type 2 diabetes mellitus, were also significantly associated with NAFLD. This concurs with several studies that have highlighted strong associations between NAFLD and hypertension, diabetes mellitus, and cardiovascular disease [11]. These associations reinforce the systemic nature of NAFLD and its extensive interplay with metabolic syndromes, emphasizing the necessity of a comprehensive approach to patient management and care. The mechanism linking NAFLD to cardiovascular disease is very complex, and both are manifestations of the end-organ damage of metabolic syndrome [11], so risk modification is recommended. Prospective studies are also needed to confirm the cause-and-effect relationship between cardiovascular disease and NAFLD.

In contrast to expectations, the FIB-4 index, a measure of liver fibrosis, did not differ between fatty-liver and non-fatty-liver patients. The FIB-4 index, as a noninvasive marker, can stratify patients with liver disease and can be used to predict liver-related morbidity and mortality [12]. It is unclear why there was no distinction between NAFLD and non-NAFLD patients among our patients. Correspondingly, further studies are needed to confirm our findings in the Saudi population.

Among the examined laboratory parameters, ALT, AST, ALP, and triglyceride levels were elevated in patients with NAFLD, indicating hepatic inflammation and metabolic abnormalities. This agrees with previous findings, signifying their importance as markers of liver injury and surrogate measures of NAFLD [13]. Additionally, global guidelines for global gastroenterology suggest that elevated ALT and AST are independent predictors of progression and mortality in NAFLD [13]. The use of liver enzymes is, therefore, crucial in both predictive and prognostic capacities in NAFLD.

Our study has several limitations. Due to its retrospective nature, we could not determine the temporality of the risk factors seen in our patients. Moreover, our patient population is largely homogenous, which may limit the generalizability of our findings. Our study did not review medications used by the cohort; this limitation can impact the findings as some medications could lead to hepatic or weight effects. Nevertheless, NAFLD studies in Saudi Arabia are limited, and our results provide valuable insights into NAFLD prediction and the risk factors associated with NAFLD for clinicians across the Kingdom.

## Conclusions

This study provides valuable insights into the role of obesity and metabolic risk factors in the development of NAFLD. The findings underscore the importance of weight management and the control of metabolic risk factors in the prevention and management of NAFLD. Further prospective studies with larger and more diverse patient cohorts are needed to corroborate and expand the findings, enabling more refined strategies for the risk prediction, early detection, and management of NAFLD.
